# Timely identification and successful treatment of acute fatty liver of pregnancy without obvious clinical symptoms: Case reports

**DOI:** 10.1097/MD.0000000000028723

**Published:** 2022-02-04

**Authors:** Weiping Cao, Tingmei Chen, Wen Jiang, Yinping Geng, Bing Xie, Qin Wang, Xinzhi Wang

**Affiliations:** a Departments of Obstetrics, Maternity and Child Health Hospital of Zhenjiang, Zhenjiang, Jiangsu, PR China; b Department of Nursing, School of Medicine, Jiangsu University, Zhenjiang, Jiangsu, PR China; c Department of Ultrasound, Maternity and Child Health Hospital of Zhenjiang, Zhenjiang, Jiangsu, PR China; d New drug screening center, Jiangsu Center for Pharmacodynamics Research and Evaluation, China Pharmaceutical University, Nanjing, PR China.

**Keywords:** acute fatty liver of pregnancy, case report, China, laboratory features

## Abstract

**Rationale:**

Acute fatty liver of pregnancy (AFLP) is a rare and potentially fatal complication that occurs in the third trimester or early postpartum period. The diagnosis of AFLP is based on typical clinical and laboratory features and imaging examinations.

**Patient concerns:**

Case 1: a 25-year-old pregnant woman was hospitalized for threatened preterm birth at gestation of 35weeks and 2 days gestation. Laboratory tests revealed liver dysfunction, coagulopathy, hypoglycemia, hypoproteinemia, leukocytosis, and elevated creatinine and uric acid levels. Case 2: a 28-year-old (nulliparous) became pregnant after in vitro fertilization-embryo transfer at 29 weeks and 1 days’ gestation and came to the obstetric ward for vaginal bleeding. At 34 weeks and 1 day, laboratory investigations showed high serum creatinine, uric acid, liver dysfunction, coagulopathy, and hypoglycemia.

**Diagnoses:**

Two patients did not show obvious clinical symptoms, while the ultrasound findings confirmed a diagnosis of AFLP.

**Interventions:**

Immediate delivery and comprehensive supportive treatment are the most important methods for the treatment of AFLP.

**Outcomes:**

The 2 patients and their babies were discharged from the hospital in a good condition.

**Lessons:**

Special attention should be paid to mothers with AFLP after in vitro fertilization-embryo transfer. The clinical presentation of AFLP is variable, hence laboratory features and ultrasound examination may be important methods for screening for AFLP.

## 1 Introduction

Acute fatty liver of pregnancy (AFLP) is an uncommon but potentially fatal disease that occurs in the third trimester or early postpartum period with microvesicular fatty infiltration of the liver, which can induce maternal multiorgan failure or even death of the mother and fetus. The approximate incidence of AFLP is 1: 7000 to 1:15,000.^[1]^ Although the incidence of AFLP is low, it remains a common cause of liver failure during pregnancy and may be accompanied by multiple organ dysfunction. The most common initial symptoms were anorexia, nausea, vomiting, abdominal pain, and jaundice. Clinical symptoms and laboratory data are key tools for the diagnosis of AFLP. Imaging may be helpful in supporting the diagnosis. Early recognition, accurate diagnosis, timely delivery, and intensive supportive care are the cornerstones of AFLP management. AFLP is an obstetric emergency; thus, early recognition and termination of pregnancy could improve its prognosis. The purpose of the 2 cases of AFLP without obvious clinical symptoms was to report our experience with early recognition and management.

## 2 Case reports

### 2.1 Case 1

**Figure 1:**
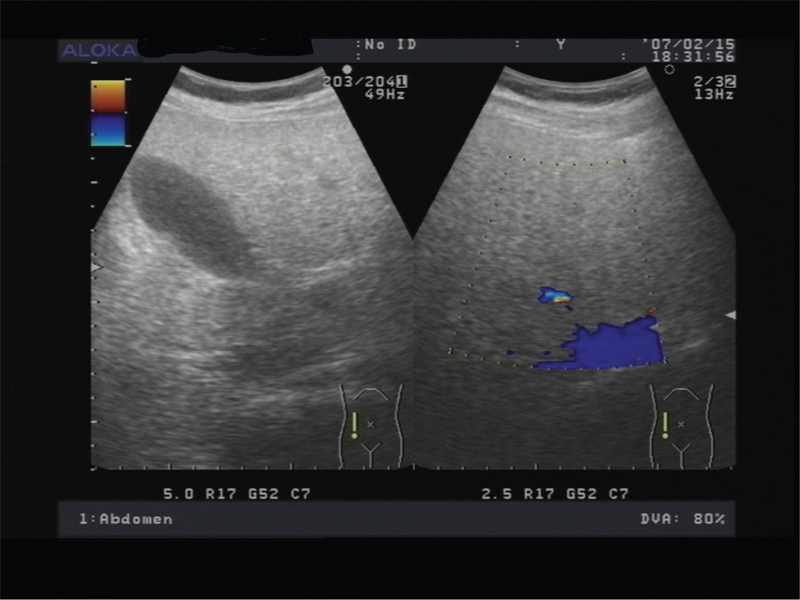
Ultrasonogram showing fatty liver. Abdominal ultrasound showed liver with an increase in echogenicity (case 1).

A 25-year-old pregnant woman in her 1st pregnancy, was hospitalized for threatened preterm birth and gestational diabetes mellitus (GDM) at 35 weeks and 2 days of gestation. On admission, the patient showed no pruritus, headache, vomiting, or other symptoms. The blood pressure, pulse, and temperature were 120/70 mm Hg, 76 bpm and 36.7°C respectively. The results of respiratory and cardiovascular examinations were normal. Her general physical examination results were unremarkable. However, blood test results were as follows: aspartate aminotransferase (AST) 140.1IU/L (reference range, 4.0–40.0 IU/L), alanine aminotransferase (ALT) 149.1IU/L (reference range, 8.0–44.0 IU/L), alkaline phosphatase 704IU/L (reference range, 40.0–150.9 IU/L), white cell count 20.44 × 10^9^/L (reference range, 3.69–9.16 × 10^9^/L), total bilirubin 86.7 mmol/L (reference range, 3.4–20.5 mmol/L), total bile acids 176.9 μmol/L (reference range, 0.0–15.0 μmol/L), serum creatinine138.9 μmol/L (reference range, 45.0–84.0 μmol/L), uric acid 459 μmol/L (reference range, 155–357 μmol/L), D-dimer of 1338 ng/mL (reference range, 0.0–255 ng/mL), activated partial thromboplastin time (APTT) of 50.0 s (reference range, 25.1–36.5), fibrinogen degradation product (FDP) 31.6 μg/mL (reference range, 0.0–5.0 μg/mL), albumin 27.6 g/L (reference range, 35.0–55.0 μg/mL), total protein 44.0 g/L (reference range, 60.0–83.0 g/L), glucose 3.79 mmol/L (reference range, 3.89–6.11 mmol/L), thrombin time (TM)16.30s (reference range, 15.8–24.9), fibrinogen of 1.79 g/L (reference range, 2.38–4.98 g/L), PT percent activity 53.0 (reference range, 80–120), antithrombin III 28% (reference range, 83–128). Biochemical results for lipids of total cholesterol, triglyceride, and low-density lipoprotein were also increased. The laboratory test results are listed in Table 1. Abdominal ultrasonography showed an increase in echogenicity in the liver (Fig. 1). The combination of laboratory findings and ultrasound findings reflecting liver compromise supported the diagnosis of AFLP, which led to the decision to terminate the pregnancy. A cesarean section was performed 1 day later after admission to deal with AFLP. On the first postpartum day, blood testing showed improvement in AST and ALT levels, but PT percent activity, D-dimer, APTT, antithrombin III, serum creatinine, uric acid, albumin, and total bilirubin remained almost the same. The newborn was male, with a birth weight of 2450 g and an Apgar score of 2 in the first minute and 6 in the fifth minute, requiring resuscitation. The newborn male was treated with suction, resuscitation, airbag pressure, and oxygen therapy. Neonatal respiratory recovery and symptoms improved. The neonate was then transferred to the neonatal intensive care unit. The patient was transferred to the hepatic department for 10 days of comprehensive treatment and eventually recovered well.

**Table 1:** Laboratory findings of case 1.

Main parameters	Admission	1Day post op	Normal range
Thrombin time (sec)	16.3	34.10	15.8–24.9
Fibrinogen (g.l^-1^)	1.79	1.64	2.38–4.98
FDP(μg/mL)	31.6	21.30	0.0–5.0
PTR ratio	1.43	1.46	0.83–2.00
PT-INR	1.42	1.45	0.82–2.00
PT percent activity	53.0	51.0	80-120
APTT (s)	50.0	45.9	25.1–36.5
Antithrombin III (%)	28	24	83–128
D-dimer	1338	1264	0–255
Total bilirubin(mmol/L)	86.7	100.5	3.4–20.5
Total protein (g/L)	58.20	43.40	60.00–83.00
Albumin (g/L)	34.90	27.40	35.00–55.00
ALT (IU/L)	149.1	86.20	8.0–44.0
AST (IU/L)	140.1	79.0	4.0–40.0
ALP (IU/L)	704	580	40–150
Hemoglobin(g/L)	112	105	113-172
Hematocrit	43.0	31.9	35.00–45.00
Platelet (× 10^9^/L)	207	144	85–320
Leucocytosis (× 10^9^/L)	20.44	27.17	3.69–9.16
Glucose (mmol/L)	3.97	4.64	3.89–6.11
Serum creatinine(μmol/L)	138.9	138.6	45.0–84.0
BUN (mmol/L)	4.49	7.58	1.43–7.14
Uric acid (μmol/L)	459	526	155–357
TBA(μmol/L)	176.9	90.1	0.0–15.0
Cholesterol total (mmol/L)	4.11	Untested	3.10–5.20
Triglyceride (mmol/L)	4.82	Untested	0.40–1.70
Low density lipoprotein (mmol/L)	3.63	Untested	0.00–3.37
High density lipoprotein (mmol/L)	0.48	Untested	1.15–2.00

The normal ranges of the variables are based on the Chinese population. AST = aspartate aminotransferase, ALT = alanine aminotransferase, ALP = alkaline phosphatase, TBA = total bile acids, FDP = fibrinogen degradation product, PT ratio = prothrombin time ratio, PT-INR = prothrombin time-international normalized ratio,. BUN = blood urea nitrogen; APTT (sec) = activated partial thromboplastin time; AFLP = acute fatty liver of pregnancy.

### 2.2 Case 2

A 28-year-old (nulliparous) became pregnant after in vitro fertilization-embryo transfer at 29 weeks and 1 days’ gestation and came to the obstetric ward for vaginal bleeding. Her respiratory rate was 18 cycles per minute, pulse was 82 beats per minute, and her blood pressure was 116/79 mm Hg. Her abdomen was uniformly enlarged, and her symphysio-fundal height was 29 cm, which was compatible with her gestational age. The fetal heart tones were regular at 140/150 beats per minute. On admission, the patient showed no pruritus, headache, vomiting, or other symptoms. The laboratory test results were normal. The lipid of cholesterol total 6.46 mmol/L (reference range, 3.10–5.20 mmol/L) and triglyceride 5.27 mmol/L (reference range, 0.40–1.70 mmol/L) and low density lipoprotein 3.70 mmol/L (reference range, 0.00–3.37 mmol/L) were also increased. Dexamethasone was administered to promote fetal lung maturation, and ritodrine hydrochloride was administered for fetal protection. The patient received continuous and routine prenatal care.

**Figure 2:**
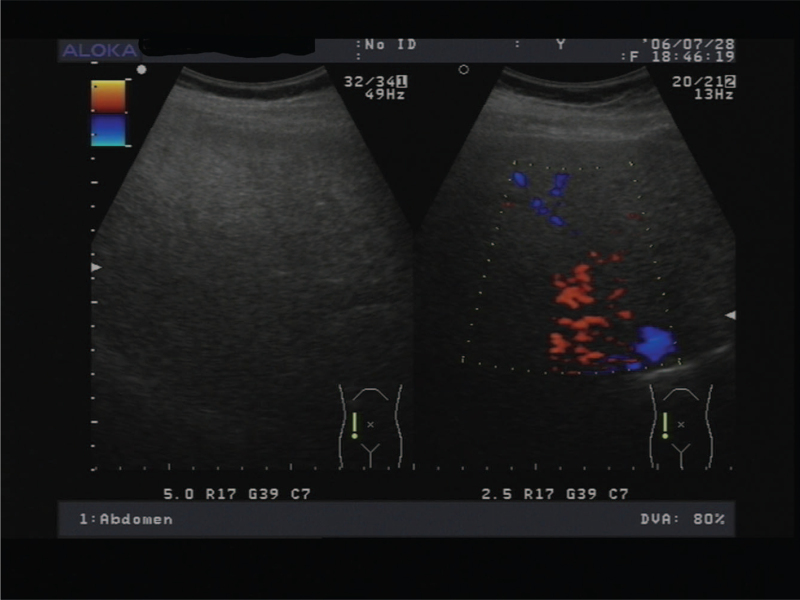
Ultrasonogram showing fatty liver. Abdominal ultrasound showed liver with an increase in echogenicity (case 2).

29 days in the hospital (34 weeks 1 day of gestation), the patient started to feel unwell. The laboratory investigations were as follows: white cell count 22.02 × 10^9^/L, ALT 336.8IU/L, AST 436.5IU/L, total bilirubin 35.2 mmol/L, total bile acid 18.8 μmol/L, serum creatinine77.2 μmol/L, uric acid 604 μmol/L, D-dimer3231 ng/mL, FDP33.0 μg/ml, APTT65.8 s, antithrombin 11136%, thrombin time11.9(s), fibrinogen 2.31(g.l-1) and glycaemia 2.76 mmol/L. The lipid of cholesterol total 2.26 mmol/L (reference range, 3.10–5.20 mmol/L) and triglyceride 1.64 mmol/L (reference range, 0.40–1.70 mmol/L) and low density lipoprotein 1.28 mmol/L (reference range, 0.00–3.37 mmol/L) were decreased (compared with admission, 6.46, 5.27, and 3.70, respectively). Table 2 presents the laboratory findings. The patient showed no clinical symptoms. Abdominal ultrasonography revealed fatty liver (Fig. 2). At this point, acute fatty liver during pregnancy was considered. In the treatment of AFLP, it is important to terminate pregnancy as early as possible to reduce the risk of severe maternal complications such as liver failure, multiple organ failure, and disseminated intravascular coagulation and increase the survival rate. Therefore, the patient underwent an emergency cesarean section. The volume of postpartum hemorrhage was approximately 1000 mL. Fresh whole blood, fresh frozen plasma, albumin, and packed cell transfusions were administered to correct bleeding. In addition to the risk of bleeding, patients are also at risk of hypoglycemia, which must be corrected. Furthermore, fetal assessment is required. Babies are twins of a male and a female, with birth weights of 1880 g and 2300 g, respectively. The Apgar scores of both infants were 9/1 min and 9/5 min. The twins were sent to NICU because of their low birth weight. The patient was closely monitored and provided supportive care in the outpatient clinic. The recovery time in this case was also the longest, and the length of hospitalization was 13 days.

**Table 2:** Laboratory findings of case 2.

Main parameters	Admission	Diagnosis	1Day post op	Normal range
Thrombin time (sec)	16.6	11.9	21.8	15.8–24.9
Fibrinogen (g.l-1)	5.52	2.31	5.20	2.38–4.98
FDP(μg/mL)	5.7	33.0	14.8	0.0–5.0
PTRratio	0.91	1.12	1.27	0.83–2.00
PT-INR	0.92	1.12	1.26	0.82–2.00
PT percent activity	107	76	63	80-120
APTT (s)	30.8	55.8	46.6	25.1–36.5
Antithrombin III (%)	92	36	49	83–128
D-dimer	978	3231	1248	0–255
Total bilirubin(mmol/L)	4.2	35.2	40.2	3.4–20.5
Total protein (g/L)	58.30	28.6	37.50	60.00–83.00
Albumin (g/L)	34.20	24.60	23.80	35.00–55.00
ALT (IU/L)	11.9	336.8	102.2	8.0–44.0
AST (IU/L)	21.2	436.5	102.0	4.0–40.0
ALP(IU/L)	137	120	108	40-150
TBA(μmol/L)	9.2	18.8	3.8	0.0–15.0
Hemoglobin(g/L)	123	123	77	113-172
Hematocrit	36.30	36.60	23.3	35.00–45.00
Platelet (× 10^9^/L)	182	84	70	100–300
Leucocytosis (× 10^9^/L)	9.97	22.02	27.43	3.69–9.16
Glucose (mmol/L)	5.42	2.76	3.26	3.89–6.11
Serum creatinine(μmol/L)	44.1	77.2	89.9	45.0–84.0
BUN (mmol/L)	2.98	7.54	8.30	1.43–7.14
Uric acid (μmol/L)	346	604	527	155–357
Cholesterol total (mmol/L)	6.46	2.26	2.15	3.10–5.20
Triglyceride (mmol/L)	5.27	1.64	1.44	0.40–1.70
Low density lipoprotein (mmol/L)	3.70	1.28	1.15	0.00–3.37
High density lipoprotein (mmol/L)	1.84	0.66	0.71	1.15–2.00

The normal ranges of the variables are based on the Chinese population. AST = aspartate aminotransferase, ALT = alanine aminotransferase, ALP = alkaline phosphatase, TBA = total bile acids, FDP = fibrinogen degradation product, PT ratio = prothrombin time ratio, PT-INR = prothrombin time-international normalized ratio, BUN = blood urea nitrogen, APTT (s) = activated partial thromboplastin time, AFLP = acute fatty liver of pregnancy.

## 3 Discussion

Early diagnosis, immediate delivery, and comprehensive supportive treatment are the mainstays of the management of AFLP.^[2]^ These 2 cases had no clinical symptoms; therefore, early identification of AFLP is difficult. Therefore, laboratory and ultrasonographic evaluations are essential. Similar to other studies, the major laboratory findings of AFLP observed in these 2 patients were liver dysfunction, coagulopathy, hypoglycemia, hypoproteinemia, leukocytosis, elevated creatinine, and uric acid. Among laboratory indicators, transaminases, serum bilirubin, leukocytosis, and coagulopathy are common.^[3]^ The 2 cases of alkaline phosphatase were also elevated, although the test was difficult to interpret at term. Leukocytosis is common in AFLP and may reach unusually high levels.^[4]^ Renal failure of variable degree is another common complication of AFLP. In the 2 cases, this may be manifested only as an elevation out of the normal range for creatinine, blood urea nitrogen, and uric acid, which was significantly higher than the normal range. Uric acid is filtered through renal glomeruli and absorbed by the first part of the proximal convoluted tubule. Hyperuricemia can lead to maternal endothelial dysfunction and affect placental blood flow. The diagnosis of AFLP can be facilitated by using the Swansea criteria (Table 3),^[5]^ which combine clinical, laboratory, and radiographic features for which the diagnosis can be made if at least 6 of the features are present. Our patient met more than 6 Swansea criteria, and so was positive. However, our case findings suggest that the currently available criteria for screening patients for possible AFLP are inadequate. For example, in these 2 cases, FDP and D-dimer levels were increased and Antithrombin 111 and fibrinogen levels were decreased, but not in the Swansea standard. While not a feature of the Swansea criteria, other studies have reported that low total cholesterol may be useful in the diagnosis of AFLP.^[6]^ Therefore, the diagnostic criteria for AFLP need to be further modified according to the clinical practice. These 2 cases show that laboratory parameters and ultrasound are very important for the diagnosis of AFLP. The most important lesson learned from the 2 cases was the timely identification of AFLP without obvious clinical symptoms.

**Table 3:** Swansea criteria for the diagnosis of acute fatty liver of pregnancy.

Variable	Finding
Vomiting	Positive
Abdominal pain	Positive
Polydipsia/polyuria	Positive
Encephalopathy	Positive
Bilirubin	> 14 μmol/L
Hypoglycaemia	< 4 mmol/L
Uric acid	> 340 μmol/L
Leucocytosis	> 11 × 106 /L
Liver ultrasonography	Ascites or bright
AST and ALT	> 42 IU/L
Ammonia	> 47 μmol/L
Creatinine	> 150 μmol/L
Coagulopathy	
PT	> 14 s
APTT	> 34 s
Liver biopsy	Microvesicular steatosis

Liver function tests also included protein and albumin levels, and clotting factors. Acute hepatic failure is the most feared catastrophe associated with AFLPs. In these 2 cases, we found that both the total protein and albumin levels decreased. Low serum albumin levels usually occur in combination with hypoproteinemia. Hypoproteinemia can lead to immune system damage, changes in colloid osmotic pressure, and coagulopathy. Acute hepatic failure and coagulopathy are also common. Such patients are likely to have low levels of fibrinogen and antithrombin 111 and raised APTT, FDP, and D-dimer-levels. Antithrombin 111 levels are low due to reduced hepatic synthesis, consumptive coagulopathy, and loss of urine proteinuria. Thus, antithrombin 111was may be helpful for the diagnosis of AFLP. D-dimer is a stable, covalently linked, dimeric fragment unique to fibrin polymerization, and degradation.^[7]^ The presence of increased D-dimer levels specifically reflects fibrin formation resulting from the activation of the coagulation cascade.^[8]^ Coagulopathy can result in excessive bleeding. Bleeding during cesarean section or vaginal hemorrhage of > 500 mL was defined as postpartum hemorrhage. The patient in case 2 had severe postpartum hemorrhage, requiring large amounts of bleeding during transfusion. The most successful treatment for this complication is replacement of clotting factors by infusion of fresh frozen plasma.

Several predisposing factors include primiparity, multiple pregnancies, male sex of the fetus, and preeclampsia.^[9]^ A primigravida carrying a male fetus and GDM (case1) was at high risk. The exact pathophysiology of AFLP is still unknown, but advanced biomolecular research indicates a defect in long chain 3-hydroxyacil coenzyme a-dehydrogenase (LCHAD). In case 2, the total cholesterol, triglyceride, and low density lipoprotein1 were higher than the normal range on admission, but lower than normal on the day of diagnosis. In recent years, studies have reported that the serum cholesterol level in patients with AFLP has decreased.^[10,11]^ However, in case 2, serum cholesterol, triglyceride, and low-density lipoprotein levels on the day of diagnosis were decreased and increased at admission with AFLP. These results indicate that lipid abnormalities are important for the diagnosis of AFLP. Two or 3 risk factors were identified in both the cases. In case 2, AFLP occurred in a twin mother after IVF embryo transfer. There is no literature on this aspect, and special attention should be paid to the mother after IVF-ET. Weaker risk factors associated with AFLP are underlying metabolic disorders, such as GDM (case1), but the cause and effect are not clear.

Early recognition, delivery, and intensive supportive care are important in the management of AFLP. Two cases were terminated by emergency cesarean delivery within 24 hours of the diagnosis of AFLP. Case 2 demonstrated abnormalities in coagulation parameters that were treated with early administration of appropriate blood products. If hypoglycemia occurs, serial glucose levels are closely monitored and corrected, if necessary. After delivery, even though the clinical and laboratory features may begin to reverse, there will be continuation of the pathophysiologic changes of AFLP for periods up to 7 to 10 days.^[12]^ Therefore, these 2 cases received supportive treatment for more than 1 week after delivery.

In conclusion, we report 2 cases of AFLP without clinical symptoms that were diagnosed based on laboratory parameters supported by ultrasonographic findings of fatty liver. AFLP is an obstetric emergency that leads to poor prognosis. However, hepatic injury associated with AFLP can be reversed if early diagnosis and termination are performed. Therefore, timely identification, early diagnosis, immediate delivery, and comprehensive supportive treatment are important for maternal and infant outcomes in patients with AFLP. Our cases show that laboratory features and ultrasound examination might be important methods for screening for AFLP.

## Author contributions

**Data curation:** Bing Xie, Qin Wang.

**Investigation:** Yingping Geng.

**Methodology:** Wen Jiang.

**Supervision:** Tingmei Chen.

**Writing – original draft:** Weiping cao.

**Writing – review & editing:** Xinzhi Wang.
